# Editorial: Molecular mechanisms of the host immune response to *Toxoplasma gondii* infection

**DOI:** 10.3389/fimmu.2025.1754559

**Published:** 2026-01-07

**Authors:** Jing Yang, Rui Fang

**Affiliations:** 1 College of Veterinary Medicine, Yunnan Agricultural University, Kunming, Yunnan, China; 2 State Key Laboratory of Agricultural Microbiology, College of Veterinary Medicine, Huazhong Agricultural University, Wuhan, Hubei, China

**Keywords:** *Toxoplasma gondii*, IFNs (interferons), IRGs (immunity-related GTPases), GBPs (guanylate-binding proteins), P2X7R, dual single-cell sequencing, scDual-Seq, ocular toxoplasmosis

Toxoplasmosis, caused by the obligate intracellular protozoan parasite *Toxoplasma gondii*, represents a significant global health concern. It poses a particularly severe threat to immunocompromised individuals and can lead to serious consequences in congenital cases. Current research has advanced our understanding of the complex interactions between different parasite genotypes worldwide and the host immune system. This Research Topic brings together six papers that collectively deepen our comprehension of the molecular antagonism mechanisms at the host-parasite interface and highlight emerging technologies and therapeutic avenues.

Lüder contributed a review summarizing the dual role of interferons (IFNs) in defense against *T. gondii*. The author elaborated on the intricate signaling networks activated by IFNs to establish cell-autonomous immunity and control parasite replication. Crucially, the review also explored the counterstrategies evolved by *T. gondii*, which utilized an array of effector proteins to disrupt these very pathways, thereby ensuring its survival and persistence. This delicate balance between host clearance mechanisms and parasite immune evasion was key to understanding infection outcomes.

Research by Singh et al. provided a detailed perspective on the determinants of virulence molecules, indicating that infection outcomes in mice were controlled by ROP5B and regulated by the interplay between host immunity-related GTPases (IRGs) and guanylate-binding proteins (GBPs). Furthermore, the study by Prado-Rangel et al. shifted the focus to a specific host receptor, P2X7R, revealing its crucial role in coordinating inflammatory responses and controlling acute infection caused by a virulent atypical strain. These findings underscored that immune efficacy was not universal but was co-determined by parasite genotype and host genetic background.

Chandrasegaran et al. performed both single-cell and bulk RNA sequencing on human peripheral blood mononuclear cells. Hildebrandt et al. employed scDual-Seq on infected mouse bone marrow-derived dendritic cells, which revealed profound heterogeneity in host cell responses and differential infection dynamics. Furthermore, the review by Chen et al. on ocular toxoplasmosis emphasized the importance of tissue-specific immunity, detailing advances in understanding local immune responses within the immune-privileged eye and discussing emerging treatment strategies aimed at preserving vision.

Overall, this Research Topic advances three pivotal themes in toxoplasmosis research. The pathogenesis of *T. gondii* stems from molecular interactions between parasite effectors and the host immune system. The strain-specific functions of ROP5 proteins and the host genotype-dependent role of the P2X7 receptor exemplify this underlying mechanistic diversity. From a methodological standpoint, the integration of single-cell and multi-omics technologies has proven indispensable. These tools are systematically uncovering hidden heterogeneity within host and parasite populations, identifying critical nodes in immune signaling networks, and revealing post-translational modifications that can modulate host responses. From a translational perspective, identifying key pathogenic determinants, essential host defense receptors, and specific cellular subpopulations that confer protection offers a new generation of potential targets for host-directed therapies and biomarker discovery.

In conclusion, this Research Topic integrates insights from parasite genetics, host immunology, and single-cell biology, propelling toxoplasmosis research further into deeper investigative realms. Future developments will involve leveraging these discoveries to devise novel interventions aimed at disrupting the delicate balance between *T. gondii* and its host, ultimately striving to alleviate the significant global burden imposed by this highly successful pathogen.

